# Retinal Vascular Changes in Radiation Maculopathy after Intravitreal Ranibizumab by Optical Coherence Tomography Angiography

**DOI:** 10.3390/jcm9061618

**Published:** 2020-05-27

**Authors:** Gilda Cennamo, Daniela Montorio, Roberta Bernardo, Antonio Farella, Raffaele Liuzzi, Maria Angelica Breve, Michele Reibaldi, Giovanni Cennamo

**Affiliations:** 1 Eye Clinic, Public Health Department, University of Naples “Federico II”, 80131 Naples, Italy; xgilda@hotmail.com; 2 Department of Neurosciences, Reproductive Sciences and Dentistry, University of Naples “Federico II”, 80131 Naples, Italy; da.montorio@gmail.com (D.M.); roberta.b.91@hotmail.com (R.B.); flangybreve@libero.it (M.A.B.); gicennam@gmail.com (G.C.); 3 Functional and Morphologic Department of Radiotherapy and Legal Medicine, University of Naples “Federico II”, 80131 Naples, Italy; antoniofarella@hotmail.com; 4 Institute of Biostructure and Bioimaging, National Research Council (CNR), 80145 Naples, Italy; raffaele.liuzzi@cnr.it; 5 Department of Surgical Sciences, University of Torino, 10124 Torino, Italy

**Keywords:** radiation maculopathy, plaque brachytherapy, optical coherence tomography angiography, retinal vessel density, foveal avascular zone

## Abstract

In this prospective study, we investigated the structural and vascular retinal changes at baseline and after Ranibizumab injections at the last follow up to one year in patients affected by Radiation Maculopathy (RM) after plaque brachytheraphy in choroidal melanoma, using Spectral Domain Optical Coherence Tomography (SD-OCT) and OCT Angiography (OCTA). A total of 40 eyes with RM of 40 patients (18 females, 22 males, mean age 51.9 ± 11 years) that underwent ruthenium-106 plaque brachytherapy were included. All patients received one monthly intravitreal injection of Ranibizumab (Pro Re Nata regimen). We analyzed the Foveal Avascular Zone (FAZ) area, the retinal vessel density (VD) of the superficial capillary plexus (SCP) and of the deep capillary plexus (DCP), using OCTA, and we detected the Central Foveal Thickness (CFT) by SD-OCT at baseline and after treatment. At the last follow up, we found a significant improvement of the CFT (*p* < 0.001) while OCTA parameters revealed no change in VD of the SCP (*p* = 0.402), DCP (*p* = 0.282), and FAZ area (*p* = 0.255), resulting in a stabilization of the visual acuity (*p* = 0.210) respect to baseline. The absence of functional improvement, despite the anatomical recovery of the macula, could be due to the absence of improvement in FAZ area and in retinal VD after treatment. OCTA parameters could represent predictive biomarkers to anti-vascular endothelial growth factor (anti-VEGF) intravitreal response and to help to better understand the physiopathological mechanisms of the RM.

## 1. Introduction

Radiotherapy is the main treatment for choroidal melanoma and, in particular, ruthenium-106 plaque brachytherapy, although it causes a local irradiation, may determine long-term damage to the retinal vascular network with the appearance of radiation maculopathy (RM), which represents a frequent complication [1,2].

The RM is a consequence of compromission of the macular microvasculature that can lead to the leakage, lipid exudates, hemorrhages, teleangiectasie, macular edema, and non perfusion areas with consequent significant impairment of visual acuity [3].

Previous evidence suggested the role of the anti-vascular endothelial growth factor (VEGF) intravitreal injections in the management of RM in light of the role of vascular permeability in the pathogenesis of macular edema [4,5].

The structural changes in the macular region after anti-VEGF treatment have been detected by the Spectral Domain Optical Coherence Tomography (SD-OCT) that it allowed to monitor the radiation-induced macular edema [6].

OCTAngiography (OCTA) is the most sensitive imaging method with which to analyze the RM because it provides detailed information on the macular microvasculature and its possible involvement on the visual acuity response after intravitreal treatment [3].

The aim of this study was to evaluate the efficacy of anti-VEGF injections on structural and vascular retinal changes in the treatment of RM, using the SD-OCT and OCTA, and the consequent influence on visual acuity.

## 2. Experimental Section

### 2.1. Subjects

In this prospective study, we enrolled a total of forty eyes of 40 consecutive patients affected by radiation maculopathy (RM) secondary to ruthenium-106 plaque radiotheraphy for choroidal melanoma treated between October 2012 and October 2019 at Eye Clinic of Federico II University (Naples, Italy).

Clinical diagnosis of RM was based on the presence of macular edema with hemorrhages, hard exudates, microaneurysms on fundus examination; cystoid macular edema and subretinal fluid on OCT; increased vascular permeability in macular region or capillary non perfusion on fluorescein angiography; and capillary loss on OCTA.

The five-point OCT-based grading scale for radiation macular edema proposed by Horgan et al. was used to characterize SD-OCT findings [7]. In this classification, Grade 1 included extrafoveolar noncystoid edema; Grade 2, extrafoveolar cystoid edema; Grade 3, foveolar noncystoid edema; Grade 4, mild-to-moderate foveolar cystoid edema; and Grade 5 included severe foveolar cystoid edema.

The eyes affected were compared with their non-irradiated fellow eyes (40 eyes) that served as a control group showing a normal ophthalmological evaluation, absence of vitreoretinal, and vascular retinal diseases.

Exclusion criteria were clinically relevant opacities of the optic media and low-quality images obtained with OCTA, presence of congenital eye disorders, pre-existing macular diseases (e.g., age-related macular degeneration, severe macular scar, or severe subfoveal exudates), pathologic myopia, history of ocular surgery, previous diagnosis of glaucoma, optic disk anomaly, and other ocular pathologic features (e.g.,vitreoretinal diseases, retinal vascular diseases).

The tumors were located at the posterior pole in 25 eyes and in retinal mid-periphery in 15 eyes.

The mean tumor thickness was measured with standardized bulbar echography before and after plaque brachytherapy. A-scan and B-scan ultrasound were performed with an AVISO-S Echograph (Quantel Medical, Clermont-Ferrand, France) and 10 and 20 MHz probes.

Radiation parameters included radiation dose (Gy) to the tumor apex, tumor base, foveal region and optic disc, time between radiotherapy and RM onset (months).

No patients showed proliferative retinopathy, neovascular glaucoma, and radiation optic neuropathy.

All patients received one monthly intravitreal injection of Ranibizumab (0.5 mg/0.05 mL) through the pars plana under aseptic conditions until maximum visual acuity was achieved and there was no sign of macular edema at structural OCT (Pro Re Nata regimen).

At baseline and after each injection, throughout the 1-year follow-up, all patients underwent complete ophthalmic examination, including best-corrected visual acuity (BCVA) according to the Early Treatment of Diabetic Retinopathy Study (ETDRS), SD-OCT, and OCTA. Fluorescein angiography (FA) and indocyanine green angiography (ICG) were performed in all patients at baseline and after reaching the recovery of visual acuity and the improvement of the retinal structures at SD-OCT. The FA was used to investigate the areas of retinal non-perfusion resulting from the radiations while the ICG was performed to evaluate, together with the ecography, the activity of the choroidal melanoma.

Two independent observers (GC; DM) carefully reviewed the SD-OCT images to evaluate Central Foveal Thickness (CFT), outer retinal layers: External Limiting Membrane (ELM) and ellipsoid zone. They investigated the OCTA images for the measurements of the Foveal Avascular Zone (FAZ) area and to analyze the vascular layers segmentation.

The study was approved by the Institutional Review Board of the University of Naples “Federico II” (NCT04310631) and all investigations adhered to the tenets of the Declaration of Helsinki. Written informed consents were obtained from the patients enrolled in the study.

### 2.2. Optical Coherence Tomography Angiography

OCTA images with the Optovue Angiovue System (software ReVue version 2014.2.0.93, Optovue Inc., Fremont, CA, USA) were performed following a standardized protocol based on the split-spectrum amplitude decorrelation algorithm (SSADA), as previously described [8].

Retinal capillary plexus was displayed performing a 6 × 6 mm scan over the macular region and the percentage area occupied by the microvasculature in the analyzed region defined the vessel density (VD) [9].

The software automatically calculates the VD in the macular scan considering several retinal vascular networks: superficial capillary plexus (SCP), deep capillary plexus (DCP). The macular region was divided in whole image, fovea, and parafovea according to the ETDRS classification of diabetic retinopathy [10]. The fovea was defined as center ring (the area within the central 1-mm ring of the ETDRS grid). Parafovea was considered as an inner ring (the area between the central 1- and the 3-mm ring of the ETDRS grid). The whole image represented the 6 × 6 mm macular area.

The FAZ area was automatically calculated by the Angiovue software (version, Manufacturer, City, US State abbrev. if applicable, Country) covering a 6 × 6 mm area over the macular region in the full retinal plexus, and it was calculated in square millimeters [11].

The software includes the 3D Projection Artifact Removal (PAR) algorithm to improve the quality of OCTA images.

Poor-quality images with a signal strength index, which reflects OCT image quality, of less than 40 or registered image sets with residual motion artefacts were excluded from the analysis.

### 2.3. Statistical Analysis

The sample size for a paired *t*-test for repeated measurements in the single group was determined from the results of our previous data to detect, with an alpha of 0.05 and 80% power, a difference of 2% in the rate of changes in retinal vessel density at OCTA and an estimated standard deviation of differences of 2. The total simple size calculated was 34 subjects and we enrolled 42 patients in case of 15% in drop out rate.

The Shapiro–Wilk test was used to test for normality and all variables were normally distributed. The paired Student’s test was used to evaluate the differences in macular VD, FAZ, CFT, and BCVA between eyes affected and their nonirradiated fellow eyes. Moreover, these parameters were analyzed at baseline and at the last follow-up after treatment. The agreement between two observers in the measurement of SD-OCT and OCTA parameters was assessed using the intraclass correlation coefficient. Statistical analysis was performed with the Statistical Package for Social Sciences (Version 20.0 for Windows; SPSS Inc, Chicago, IL, USA). A *p* value < 0.05 was considered statistically significant.

## 3. Results

Overall, 42 patients were enrolled in this study, for which two patients were excluded because they were lost to follow up. A total of forty eyes with RM of 40 patients (18 females, 22 males, mean age 51.9 ± 11 years) were included in this study.

According to the OCT-based grading scale, 10 patients presented the Grade 3 radiation macular edema, 22 patients presented the Grade 4 and 10 patients the Grade 5.

At baseline, the mean tumor thickness was 3.22 ± 0.5, the mean calculated irradiation was 100 Gy at the tumor apex and 152.93 ± 53.85 Gy (range 123.61 ± 204.41 Gy) at base. The mean distance from the fovea was 9.1 ± 2.1 mm (range 7–12.5 mm), without macular and juxtapapillary tumors.

The mean radiation dose to the fovea was 11.73 ± 16.39 Gy (range, 1.17–43.52 Gy) and to the optic disc was 9.68 ± 9.10 Gy (range, 0.15–24.29 Gy). The mean time between the brachyteraphy and the RM diagnosis was 1.9 ± 0.3 years and all patients received a mean of 7 ± 1 intravitreal injections of Ranibizumab during a follow up of 11 ± 2 months (Table 1).

The agreement between two observers for measuring the SD-OCT and OCTA parameters was excellent, with an intraclass correlation coefficient of 0.93.

As shown in Table 2, the OCTA exam showed in eyes with RM at baseline compared to their nonirradiated fellow eyes a statistically significant reduction in VD of SCP in whole image (44.03 ± 2.43% vs. 50.47 ± 3.28%; *p* < 0.001), as well as in inner ring (46.36 ± 2.34% vs. 52.27 ± 3.80%; *p* < 0.001) and in center ring (24.12 ± 3.03% vs. 33.17 ± 3.73%; *p* < 0.001). A statistically significant reduction in VD was also evident in DCP in whole image (47.15 ± 3.03% vs. 56.72 ± 3.59%; *p* < 0.001), inner ring (49.66 ± 3.59% vs. 58.02 ± 3.46%; *p* < 0.001), and center ring (31.09 ± 2.58% vs. 41.37 ± 3.87%, *p* < 0.001).

**Figure 1:**
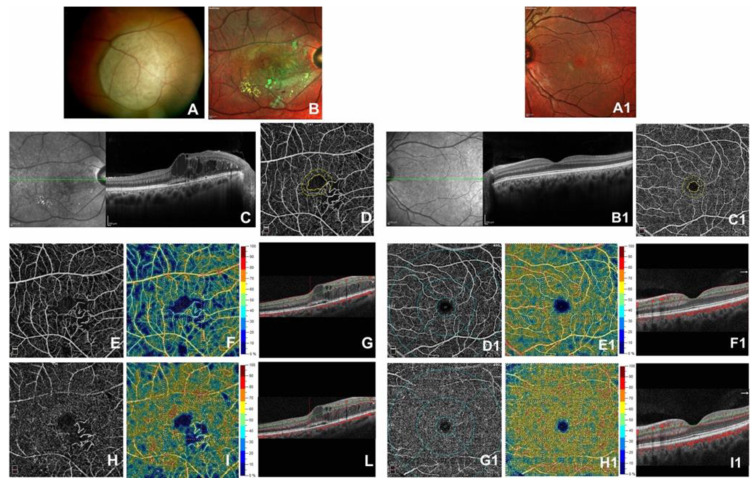
Right eye of a 37-year-old patient affected by Radiation Maculathy before Ranibizumab injections (left side of the panel) and unirradiated fellow eye (right side of the panel). Color fundus image showed in the right eye an elevated and yellow lesion in nasal mid-peripheral of the retina (**A**). Multicolor image showed large lipidic exudation and intraretinal hemorrhages at the posterior pole in eye affected with (**B**) respect to the fellow eye that presented a normal multicolor image (**A1**). Spectral Domain-Optical Coherence Tomography (SD-OCT) B-scan revealed in the right eye an increased central foveal thickness, intraretinal cysts with cystoid macular edema and a slight subfoveal exudative detachment with (**C**) respect to the fellow eye that did not present alterations of the retinal layers architecture (**B1**). At OCT Angiography (OCTA) images in macular region of the eye affected, Foveal Avascular Zone (FAZ) area appeared increased (**D**) while the Superficial Capillary Plexus and Deep Capillary Plexus presented a capillary rarefaction (**E**,**H**) and a reduction in vessel density (**F**,**I**). OCTA revealed a normal FAZ area (**C1**) and an absence of changes in retinal vascular networks (**D1**,**G1**) and vessel density (**E1**,**H1**). OCTA B-scan showed the segmentation of the retinal plexuses in both eyes (**G**,**L**,**F1**,**I1**).

While a significant enlargement of the FAZ area (0.341 ± 0.07 mm² vs. 0.211 ± 0.04 mm²; *p* < 0.001) was found in eyes affected with respect to their fellow eyes, a statistically significant increase in CFT (517.40 ± 43.96 µm vs. 264.05 ± 49.27 µm; *p* < 0.001) and a worse visual acuity (0.41 ± 0.08 LogMAR vs. 0.10 ± 0.08 LogMAR; *p* < 0.001) were found in eyes affected with respect to their fellow eyes (Figure 1).

When comparing the eyes with RM before and at the last follow up after the treatment, there were no statistically significant differences in VD of SCP and DCP in different macular regions (*p* > 0.05). The FAZ area was slightly increased, but the difference was not statistically significative (0.341 ± 0.07 mm² vs. 0.354 ± 0.04 mm²; *p* = 0.255).

**Figure 2:**
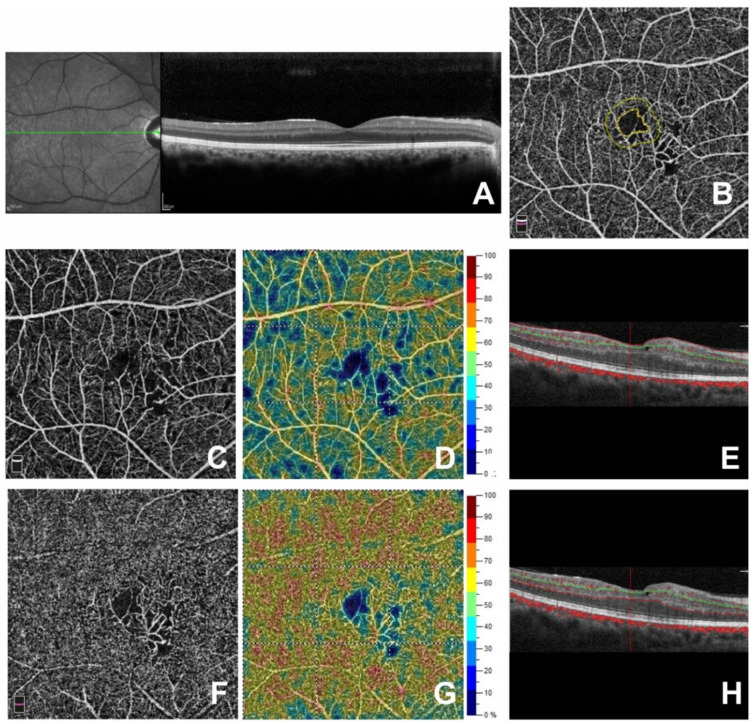
Right eye of a 37-year-old patient affected by Radiation Maculathy after Ranibizumab injections at the last follow up to one year. (**A**) Spectral Domain-Optical Coherence Tomography (SD-OCT) B-scan revealed a decreased central foveal thickness, absence of intraretinal cysts, and subfoveal exudative detachment. In OCT Angiography (OCTA) images in the macular region, no change was found in the Foveal Avascular Zone area with (**B**) respect to baseline (Figure 1), the capillary rarefaction persisted in the Superficial Capillary Plexus and Deep Capillary Plexus (**C**,**F**) with a decreased vessel density (**D**,**G**). OCTA B-scan showed the segmentation of the retinal plexuses (**E**,**H**).

Regarding the structural OCT, the integrity of the outer retinal layers (ELM and the ellipsoid zone) and the statistically significant reduction of CFT, due to reabsorption of subretinal fluid (517.40 ± 43.96 µm vs. 265.7 ± 31.55 µm; *p* < 0.001), were observed after the intravitreal injections even if the reduced visual acuity did not improve significantly (0.41 ± 0.08 LogMAR vs. 0.42 ± 0.07 LogMAR; *p* = 0.210) (Table 3 and Figure 2).

**Figure 3:**
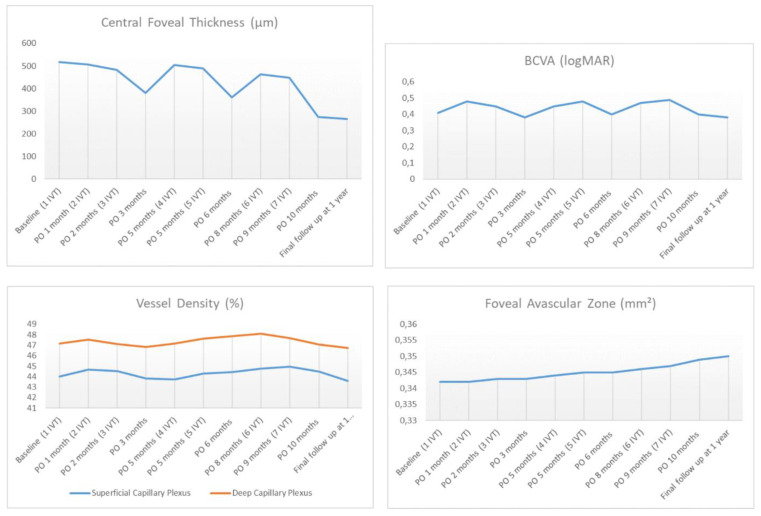
Graphs of measurements of the central foveal thickness (CFT), best corrected visual acuity (BCVA), the retinal vessel density, and the foveal avascular zone in each post operative (PO) intravitreal injection during 1 year follow up. The CFT revealed a reduction at the final follow up with respect to baseline while the BCVA and the OCTA parameters did not show any significant change during follow up.

Schematic graphs showing the measurements of SD-OCT, OCTA parameters and BCVA after each intravitreal injection during a 1 year follow up were reported in Figure 3.

## 4. Discussion

To our knowledge, this is the first study that investigated the structural and vascular retinal changes in eyes affected by RM before and after anti-VEGF intravitreal injections.

The RM represents the main complication occurring in brachytherapy, due to a irradiation-induced microangiopathy [12].

The radiation causes DNA damage of the vascular endothelial cells that determine an increased cellular proliferation, partly induced also by VEGF, with consequent progressive capillary occlusion and ischemia [7,12,13].

The VEGF also plays a role in the vascular permeability resulting in the appearance of macular edema, lipid exudation, intraretinal hemorrhages, cotton wool spots, mycroaneurisms, and telangiectasia [14,15,16].

The RM is an occlusive retinal microangiopathy, responsible for impaired visual acuity, that found in the vascular permeability a crucial role in the pathophysiological mechanisms; therefore, the use of anti-VEGF agents proved to be useful in the treatment of this condition [4].

It is important to use SD-OCT and OCTA, highly sensitive technologies, that, performing a detailed and quantitative analysis, may represent helpful biomarkers to predict the efficacy of anti-VEGF injections.

Our results demonstrated in eyes with RM with respect to nonirradiated fellow eyes a significant increase in CFT and FAZ area, a reduction in retinal VD, and an impaired visual acuity, as confirmed by previous reports [4,17,18,19,20,21]. The decreased VD in retinal vascular networks could be due to a retinal ischemia, following brachytherapy, or to the presence of macular edema. The intraretinal cystoid spaces could physically displace the retinal capillaries into adjacent layers or the intraretinal fluids could determine the attenuation of decorrelation signal at OCTA. It is also possible that the mechanical pressure by the intraretinal cystoid spaces could cause a capillary narrowing or occlusion [22].

At last follow up after treatment to one year, we found a significant decrease of the CFT, due to the reabsorption of subretinal fluid, while OCTA parameters revealed no significant change in retinal VD and a persistent increased FAZ area resulting in a stabilization of the visual acuity with respect to baseline. These findings demonstrated that the persistent reduction of VD, despite the improvement of the CFT, was related to a true absence of flow due to retinal vascular damage rather than the mechanical effect of intraretinal cystoid edema.

Previous studies have performed only a morphological analysis by SD-OCT showing a retinal structural recovery and a functional stabilization or improvement [1,23,24,25,26,27,28].

Finger et al. reported long-term experience for RM treated by Bevacizumab and Ranibizumab injections every 4 or 12 week intervals, showing at last follow up to 10 years a thinning of the CFT. BCVA was stable or improved while few cases revealed loss of more than three lines from their baseline [23]. Similar results were demonstrated by Fallico and Murray that evaluated the efficacy of intravitreal Aflibercept over the 24 months [1,24].

In addition, the use of intravitreal dexametasone implant for RM showed an anatomical recovery even if it was not correlated with a significant improvement in visual acuity [25,26]. Shields et al. indeed demonstrated that the intravitreal triamcinolone acetonide stabilized or improved the BCVA, but its effect decreased slowly after six months [27].

Conversely, Russo et al. demonstrated the efficacy of the intravitreal Dexametasone implant and Ranibizumab in anatomical and functional improvements after 24 months [28].

This low and variable visual recovery has been supposed by the authors as a consequence of the radiation-induced damage to the retinal structures such as photoreceptors and retinal pigment epithelium or due to a prolonged status of macular edema [25,26].

The analysis of the OCTA parameters could also be helpful to evaluate the influence of the retinal vascular network to the anatomical and functional outcomes after anti-VEGF treatment to better understand its efficacy.

Daruich et al. reported a progressive and significant visual acuity loss and FAZ enlargement over a six-month period during Ranibizumab or Bevacizumab intravitreal injections [2].

These results were less evident in patients with strict adherence to treatment protocol (2 month-interval) than those with variable intervals of treatment and those without treatment, while the significant improvement in central macular thickness was observed in both treated groups.

Moreover, a moderate and significant correlation between BCVA change and FAZ change over six months has been found [2].

Our results reported, although a thinning of the CFT, no improvement of the BCVA, an impairment of the FAZ area, and no change of the retinal VD over 12 months of follow up after treatment, administered monthly, with respect to baseline.

According to a study conducted by Matet et al, the worse visual acuity, induced by the RM, would seem to be influenced not only by the retinal structural factors but mostly by the vascular factors, including FAZ area and retinal VD. They confirmed, using a multivariate analysis, that larger FAZ area and lower deep plexus capillary density had a negative influence on BCVA [20].

The role of the retinal vascular involvement on the visual function was demonstrated by Shields and Say that found in irradiated eyes, also in absence of clinical evidence of RM, a significantly larger FAZ area and a reduction in parafoveal capillary density (superficial and deep), a sign of a subclinical microvascular ischemia, that caused an impaired visual acuity [19,29].

Among the treatments for RM, anti-VEGF injections, administered at regular intervals, allowed anatomical benefits, but variable results have been proven regarding the preservation of BCVA [2].

We hypothesized that the visual acuity stabilization, despite the reabsorption of subretinal fluid and the integrity of the outer retinal layers, could to be due to the absence of improvement in FAZ area and in retinal VD after treatment because the anti-VEGF agents, acting on the vascular permeability, would influence the retinal exudation rather than the vascular blood flow.

Therefore, in RM, being an occlusive microangiopathy, the FAZ enlargement and the decreased perfusion in superficial and in particular deep plexuses would be responsible for a worse visual acuity [30,31,32,33].

Further longitudinal studies with a wider sample of patients are needed to assess whether other variables such as the radiation dose on the fovea, the pattern of the macular edema or the status of impaired retinal perfusion at baseline could influence the anti-VEGF response.

The limitation of this study concerns the 3D PAR algorithm of the OCTA that did not always remove the projection artifacts. Some en face DCP images presented a partial improvement.

## 5. Conclusions

In conclusion, our study provided detailed analysis about the retinal vascular and structural features in RM induced by ruthenium -106 plaque brachytherapy over 12 months of follow up after Ranibizumab treatment suggesting that OCTA parameters could represent predictive biomarkers to anti-VEGF intravitreal injections regarding the anatomical and functional response.

## Figures and Tables

**Table 1:** Demographic characteristics, radiation parameters, number of injections and follow up in patients with radiation maculopathy.

Eyes (*n*)	40
Sex (Male/Female)	22/18
Age (years; mean ± SD)	51.9 ± 11
Tumor Thickness before brachytheraphy (mm)	3.22 ± 0.5
Tumor Thickness 1 months after brachytheraphy (mm)	1.87 ± 0.5
Mean Radiation Zone (Gy)	
Tumor apex	100
Tumor base	152.93 ± 53.85
Fovea	11.73 ± 16.39
Optic disc	9.68 ± 9.10
Time plaque-RM (years)	1.9 ± 0.3
Intravitreal injections (n.)	7 ± 1
Follow up (months)	11 ± 2

Data expressed as mean ± SD; Gy: Gray; RM: radiation maculopathy; SD: standard deviation.

**Table 2:** Differences in OCT angiography vessel density, central foveal thickness, and BCVA between the eyes with radiation maculopathy (before intravitreal injections) and their fellow eyes.

	Eyes with RM	Fellow Eyes	*p*-Value
**OCT Parameters (%)**			
Superficial Capillary Plexus			
Whole	44.03 ± 2.43	50.47 ± 3.28	<0.001
Parafovea (Inner ring)	46.36 ± 2.34	52.27 ± 3.80	<0.001
Fovea (Center ring)	24.12 ± 3.03	33.17 ± 3.73	<0.001
Deep Capillary Plexus			
Whole	47.15 ± 3.03	56.72 ± 3.59	<0.001
Parafovea (Inner ring)	49.66 ± 3.59	58.02 ± 3.46	<0.001
Fovea (Center ring)	31.09 ± 2.58	41.37 ± 3.87	<0.001
Foveal Avascular Zone (mm²)	0.341 ± 0.07	0.211 ± 0.04	<0.001
CFT (µm)	517.40 ± 43.96	264.05 ± 49.27	<0.001
BCVA (logMAR)	0.41 ± 0.08	0.10 ± 0.08	<0.001

Data expressed as mean ± SD; RM: Radiation Maculopathy; OCT: Optical Coherence Tomography CFT: Central Foveal Thickness; BCVA: Best Corrected Visual Acuity; logMAR: logarithm of the minimum angle of resolution. The paired Student’s test, *p* < 0.05.

**Table 3:** Differences in OCT angiography vessel density, central foveal thickness and BCVA before and after intravitreal injections in eyes with radiation maculopathy.

	Baseline	1-Year Follow Up	*p*-Value
**OCT Parameters (%)**			
Superficial Capillary Plexus			
Whole	44.03 ± 2.43	43.58 ± 2.85	0.402
Parafovea (Inner ring)	46.36 ± 2.34	46.03 ± 2.73	0.363
Fovea (Center ring)	24.12 ± 3.03	23.89 ± 2.93	0.740
Deep Capillary Plexus			
Whole	47.15 ± 3.03	46.75 ± 3.24	0.282
Parafovea (Inner ring)	49.66 ± 3.59	49.71 ± 3.37	0.935
Fovea (Center ring)	31.09 ± 2.58	30.12 ± 2.83	0.109
**Foveal Avascular Zone (mm²)**	0.341 ± 0.07	0.354 ± 0.04	0.255
**CFT (µm)**	517.40 ± 43.96	265.7 ± 31.55	<0.001
**BCVA (logMAR)**	0.41 ± 0.08	0.42 ± 0.07	0.210

Data expressed as mean ± SD; CFT: Central Foveal Thickness; BCVA: Best Corrected Visual Acuity; logMAR: logarithm of the minimum angle of resolution. The paired Student’s test, *p* < 0.05.
